# Cross-talk between redox signalling and protein aggregation

**DOI:** 10.1042/BST20190054

**Published:** 2020-04-20

**Authors:** Loes van Dam, Tobias B. Dansen

**Affiliations:** Center for Molecular Medicine, Molecular Cancer Research, University Medical Center Utrecht, Universiteitsweg 100, 3584CG Utrecht, The Netherlands

**Keywords:** amyloid, protein aggregation, reactive oxygen species, redox signalling

## Abstract

It is well established that both an increase in reactive oxygen species (ROS: i.e. O_2_^•−^, H_2_O_2_ and OH^•^), as well as protein aggregation, accompany ageing and proteinopathies such as Parkinson's and Alzheimer's disease. However, it is far from clear whether there is a causal relation between the two. This review describes how protein aggregation can be affected both by redox signalling (downstream of H_2_O_2_), as well as by ROS-induced damage, and aims to give an overview of the current knowledge of how redox signalling affects protein aggregation and vice versa. Redox signalling has been shown to play roles in almost every step of protein aggregation and amyloid formation, from aggregation initiation to the rapid oligomerization of large amyloids, which tend to be less toxic than oligomeric prefibrillar aggregates. We explore the hypothesis that age-associated elevated ROS production could be part of a redox signalling-dependent-stress response in an attempt to curb protein aggregation and minimize toxicity.

## Introduction

Both the loss of proteostasis and ROS production as a consequence of mitochondrial dysfunction are among the Hallmarks of Ageing [1]. While there is plenty of evidence that these two hallmarks are tightly intertwined, their cause and effect relationships remain unclear. This might be partly due to the fact that ROS, in the form of H_2_O_2_, itself plays a dual role. While at lower levels H_2_O_2_ acts as a second messenger in redox signalling, which is absolutely required for physiology and for lifespan extension in model systems [2], at higher levels H_2_O_2_ and other ROS could lead to random damage including for instance protein unfolding and aggregation, and the latter has been proposed to accelerate the aging process [3–10]. But there is also evidence of functional redox signalling-dependent protein aggregation, for instance providing a means to (temporarily) inactivate or alter the function of proteins. Redox-dependent protein aggregation is also often reversible. But there are also many examples of protein aggregation-induced enhanced ROS production, which may eventually contribute to cellular dysfunction and cell death.

A hypothesis that could unite both H_2_O_2_ as a signalling molecule and ROS as a driver of age-related protein aggregation was posed by Hekimi et al. [2]. It proposes that age-related damage triggers stress response pathways that could depend on redox signalling and hence produce H_2_O_2_ (either directly or indirectly through O_2_^•−^ followed by dismutation) as a second messenger in an attempt to regain homeostasis. Over time, when more damage accumulates, H_2_O_2_ produced to further boost this stress response surpasses levels that are merely involved in signalling, leading to a build-up of H_2_O_2_ and ROS associated damage. This eventually would lead to a vicious cycle in which ROS-dependent damage triggers a redox signalling-dependent-stress response, leading to a further increase in ROS. This hypothesis would also fit with the observations that several types of aggregates can trigger ROS production.

In this review, we will focus on examples of the interplay between redox signalling and protein aggregation. We review the current knowledge and try to illuminate possible relationships between redox signalling and proteostasis.

### Protein folding

Proteins are synthesized as linear peptide chains on ribosomes and must fold into 3D structures to execute their biological functions. Protein folding is driven for a large part by the spontaneous burial of nonpolar amino acids in the folding core, but also guided by hydrogen bonds, van der Waals- and electrostatic interactions. The many weak, noncovalent and often distant (in sequence) interaction possibilities complicate the conformational possibilities. The stability of natively folded proteins depends on local environmental factors such as pH and temperature.

Most (∼70%) protein folding takes place at the ribosome during translation. Several mechanisms are in place to ensure correct folding: the sequential folding of domains emerging from the ribosome, the spatial restrictions of the ribosomal exit channel, the rate of translation as well as the ribosome-associated chaperones RAC and NAC (ribosome-associated complex and nascent-chain associated complex, respectively) [11,12]. More downstream of the ribosome, the HSP70 system of chaperones prevents undesirable domain interactions. Moreover, HSP70 functions as a binding interface for other chaperones like HSP90 and chaperonins, which aid in *de novo* folding by recognizing exposed hydrophobic residues and promoting ATP-dependent refolding.

**Figure 1.**
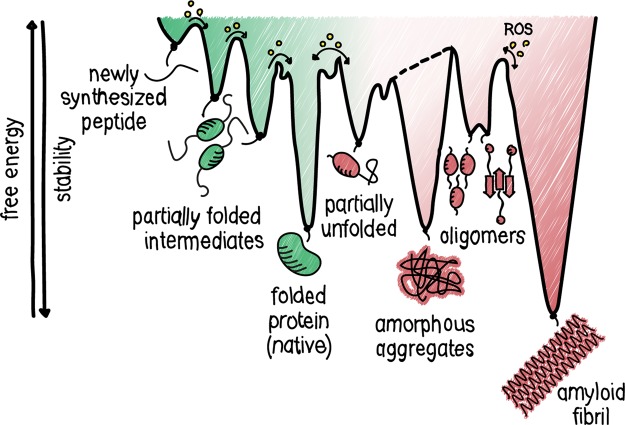
Energy landscape in proteostasis. Newly synthesized peptides sample different conformations during protein folding, on their way downhill to the most thermodynamically favourable state. Energetically trapped, partially unfolded or sub-optimally folded intermediates may accumulate as they need to cross energy barriers to reach their native, low energy state. Non-native interactions may lead to protein aggregation, thereby interfering with the protein folding process. The proteostasis network can assist in lowering energy barriers and preventing non-native interactions. As indicated by the yellow circles, H_2_O_2_-mediated redox signaling or ROS-dependent damage helps to overcome the transition state between intermediates of the proteostasis network.

Proteins need to overcome considerable energy barriers to reach their final, stable conformation, inherently leading to the accumulation of folding intermediates (Figure 1) [13]. Examples of slow steps in protein folding include disulfide bond formation and prolyl isomerization [14]. Partially folded proteins are at high risk of misfolding and aggregation, due to non-native interactions through for instance the exposure of hydrophobic residues [15,16]. Other reasons for faulty protein accumulation are mutations or polymorphisms, translation errors and the structurally dynamic characteristic of proteins. Misfolded conformations are quasi-stable, making them more prone to aggregation when proteostasis control systems are saturated. The latter happens increasingly during ageing [17]. The high plasticity of partially folded intermediates is in contrast with extremely structured aggregates like amyloid fibrils.

Another hallmark of ageing, the failure to maintain proteostasis, presents itself as an accumulation of misfolded proteins and aggregates. Like folding, aggregation is predominantly driven by hydrophobic interactions, which is why aggregation prone regions (APRs) in proteins are generally distinguished by their high hydrophobicity, low net charge and high β-sheet propensity [18,19]. During aggregation, the hydrophobic interactions are mostly intermolecular, and therefore aggregation is concentration dependent. Due to the similarity between the composition of APRs and protein regions driving hydrophobic core formation during folding, aggregation and folding pathways constantly compete. The aggregation has long been considered only as a sign of degeneration and dysfunction. However, despite strong selective pressure against protein aggregation, numerous APRs remain in the proteome, which is in line with the notion that protein aggregation can play a functional or regulatory role [20,21]. The number of possible conformations for aggregation intermediates is large, and they need to overcome free energy barriers on their way to mature aggregates. This means that aggregation intermediates are energetically trapped and thus accumulate (Figure 1), allowing more non-native interactions to occur. Typically, these non-specific interactions between polypeptides form a disordered assembly without a specified shape termed amorphous aggregates.

While most aggregates are amorphous, examples of more structured aggregate types are oligomeric aggregates and the extremely structured β-amyloid fibrils, and the latter are characterized by a cross-β-structure (in which β-strands lie perpendicular to the fibril axis). Amyloid fibrillization consists of a slow lag phase directed by intermolecular interactions during which misfolded polypeptides congregate into nuclei and form oligomers containing β-sheets. During the subsequent exponential growth phase, oligomers cluster further with these nuclei and grow rapidly into prefibrils and protofibrils with a cross-β-structure. During the final saturation phase, 2–6 protofibrils assemble into mature multistrand amyloid fibrils which can adopt several polymorphic structures by twisting or lateral association [22]. Amyloid fibrils are one of the most thermodynamically stable and stiff protein arrangements known [23].

Amyloids are a hallmark of (age-related) proteinopathies such as ALS, Alzheimer's, prion disease and cataract. Early studies focused on the mature aggregate deposits as toxic species. The toxicity of amyloids is complex, however, and several intermediates and oligomers but also mature amyloid fibrils have now been linked to pathogenesis [11–13]. Some reports even suggest an inverse correlation between oligomer size and toxicity of aggregates [24–27]. This complexity is also thought to be one of the reasons for clinical trial failures in proteinopathies, with hardly any effective therapy available for treatment [13,28]. Adding to the complexity is the fact that many types of amyloids are shown to be functional with roles reported in chemical storage, structure, signalling and inactivation of the soluble protein [29]. Another type of functional aggregation is controlled by protein domains lacking rigid 3D structures under physiological conditions, present in 15–30% of proteins. These intrinsically disordered regions (IDRs) have a high conformational plasticity and susceptibility to modifications, enabling a multitude of interactions [30]. With these interactions, IDRs drive misfolding and aggregation of (partially) disordered proteins as well as liquid–liquid phase separation (LLPS), thereby forming membrane-less compartments which are important for the concentration and segregation of biochemical reactions [31]. However, LLPS condensates have also been reported to catalyze amyloid fibrillization [32,33].

### Clearance of protein aggregates

There are three quality control networks to ensure continuous surveillance of the proteome: (i) chaperones that mediate (re)folding, (ii) the ubiquitin-proteasome system (UPS) and (iii) autophagy to clear misfolded proteins and aggregates.

As mentioned previously, chaperones aid in *de novo* protein folding by lowering energy barriers between folding intermediates. During aggregation, chaperones instead raise the energy barriers toward aggregation by preventing intermolecular interactions. Besides preventing aggregation, chaperones play an important role in active disaggregation [34]. Recent reports show that almost all types of aggregates are reversible [35,36]. The co-ordinated action of small HSPs (sHSPs), HSP40, HSP70 and HSP110 can even disaggregate amyloid fibrils by fragmentation and depolymerization into both monomeric and oligomeric species. The activity of chaperones is, therefore, twofold: on the one hand to prevent aggregation, on the other to disaggregate (intermediate) aggregates.

One of the major protein degradation systems is the UPS. There are several constitutions of the proteasome, but the 20S and 26S are most prominent. Ubiquitinated proteins and insoluble aggregates are pulled through the proteasomal ring-like structure, to be broken down by proteolysis. Not surprisingly, proteasomal dysfunction is associated with ageing and leads to the accumulation of aggregates [5–7,9,10], catalyzing a chain reaction in which aggregates block the proteasome which causes further dysfunction [37].

A third mechanism by which cells can clear and recycle cellular components is by autophagy. Many types of protein aggregates are cleared by autophagy, including τ, Aβ, α-synuclein, huntingtin, SOD1 and p16^INK4A^ [38–43], with autophagy defects leading to neurodegenerative disease [44,45].

Defects in and decreased activity of any of these proteostasis surveillance systems are associated with ageing and proteinopathies, underpinning their importance in maintaining proteostasis.

### Redox signalling

Increased markers for ROS-induced damage and mitochondrial ROS generation have long been associated with age and neurodegenerative disorders, which has often been regarded as evidence for a causal link between oxidative damage and ageing [46–54]. This is in contrast with the unexpected observation that increased ROS levels may extend lifespan in yeast and *Caenorhabditis elegans* [55,56], while increased ROS does not accelerate ageing in mice [57]. The key to resolve this apparent paradox probably lies in the notion that ROS in the form of H_2_O_2_ has become widely recognized as the second messenger in so-called redox signalling, which has been shown to be involved in a plethora of cellular responses [46]. Central to redox signalling is the reversible oxidation/reduction of the nucleophilic thiol side chain of specific cysteine residues. Oxidation of cysteines in proteins commonly causes structural changes and functional interactions through disulfide bond formation, such as (hetero)dimerization, oligomerization, and even aggregation, and thereby provides an important molecular switch for protein activity or function. For a comprehensive review see [58].

Compared with superoxide anions and hydroxyl radicals, the other main cellular ROS, H_2_O_2_, has a relatively low reactivity and allows for specific rather than random oxidation of dedicated cysteines. Redox signalling starts with the production of H_2_O_2_: either directly (i.e. by DUOX enzymes or ERO1-dependent protein folding in the ER) or after dismutation of superoxide produced by the leakage of electrons from complex I and III of the electron transport chain during mitochondrial respiration, or by NADPH-dependent oxidases (NOXs). For a comprehensive review on subcellular sources of ROS see [59].

Superoxide and H_2_O_2_ are efficiently scavenged by antioxidant systems and the balance between ROS production and antioxidant capacity determines the redox state of cellular compartments [60,61]. When the amount of ROS surpasses the levels required for signalling, non-specific cellular damage (oxidative stress) can occur due to their high reactivity with biomolecules. Furthermore, H_2_O_2_ may be converted by more reactive species in the presence of transition metal ions, thereby changing its role from messenger to damaging agent. In line with this, it has been suggested that oxidative damage exists merely as a side product of cellular signalling, and that ROS are in fact part of the stress response to for instance proteotoxic stress [2,62]. A good distinction between ROS as a signalling molecule versus ROS as oxidative stress has been proposed by Sies et al. [59]. Throughout this review, we will try to use the term ‘ROS’ in case the exact species is unclear or in the case of random damage rather than signalling, whereas redox signalling and reversible cysteine oxidation are considered regulated processes downstream of H_2_O_2_.

Other amino acid side chains besides cysteine and methionine are also subject to oxidation. For example, Tyr and Trp phenoxyl radicals, carbonylated Lys/His/Cys, SNO-/SSG-modifications as well as lipid peroxidation and its aldehyde byproduct 4-hydroxynonenal (HNE) are all ROS-induced and have been associated with proteinopathies [63,64]. Collectively, the dual roles of H_2_O_2_/ROS in signalling and damage make it difficult to understand whether the increase in ROS in ageing represents a causal link with oxidative damage, redox signalling or both. In this review, we will focus mostly on the effects of reversible cysteine oxidation-dependent redox signalling but will also include examples of how random oxidative damage affects protein aggregation.

## Redox signalling and protein aggregation

Protein aggregation can be affected or directly regulated by redox signalling or the cellular redox state. In general, cysteine oxidation results in structural changes, for instance through disulfide formation, that affect protein function [58]. These structural changes can also provide a molecular switch to partially unfold and subsequently aggregate. Interestingly, many proteins are predicted to contain conditionally disordered regions that could be redox sensitive and thereby facilitate the transition from disorder-to-order or order-to-disorder dependent on oxidation or reduction[65]. Similar to IDPs, this transition is driven by the multiplicity of possible interactions. Conversely, it is also known that misfolded proteins are more sensitive to oxidation, which has been suggested to tag proteins for proteolysis [66].

### Functional redox-dependent aggregation

As mentioned earlier, several amyloid fibrils have functional roles in humans. Amyloid fibril formation can also be reversible [67,68] and reversible oxidation of cysteines can provide the molecular switch that regulates fibrillization. For example, the tumour suppressor p16^INK4A^ is readily oxidized on its only cysteine in an oxidizing environment, causing disulfide-linked homodimerization and subsequent rapid but reversible β-amyloid fibrillization. This redox-dependent change from a soluble monomeric protein into insoluble but reversible β-amyloid fibrils leads to the inactivation of the protein, allowing reactivation of CDK4/6 proteins otherwise inactivated by high p16^INK4A^ expression [69]. These observations fit with the notion that redox signalling can regulate S-phase entry and cellular proliferation [70,71].

Another illustration of redox-dependent functional protein aggregation is provided by tryptophan hydroxylase 2 (TPH2), the rate-limiting enzyme in serotonin neurotransmitter production. TPH2 aggregates upon oxidation of any out of 13 cysteines and subsequent intra- and intermolecular disulfide bond formation, thereby reversibly inhibiting protein activity. TPH2 catalytic activity correlates directly with the number of cysteines that are oxidized [72,73]. Although the direct purpose for redox regulation of serotonin biosynthesis is unknown, it provides a link between neurological function and redox signalling, a concept that is widely accepted in the molecular regulation of circadian rhythm [74].

In a similar way, redox status is linked to intracellular calcium concentrations through oxidation-dependent protein aggregation of visinin-like protein-1 (VSNL1), a neuronal calcium sensor important that can activate guanylyl cyclase (GC). Under low calcium concentrations, GC produces the second messenger cGMP that can activate calcium channels. When bound to calcium, structural rearrangements in VSNL1 make the C-terminal C187 available for reversible oxidation and subsequent homodimerization and aggregation. These disulfide cross-linked aggregates are reversible upon treatment with reducing agents. As a result of aggregation, functional levels of VSNL1 are decreased, possibly providing a second mechanism to keep GC inactive besides calcium levels. Furthermore, VSNL1 aggregates are found in amyotrophic lateral sclerosis (ALS)-associated deposits, linking them to neuronal impairment [75–79].

Conversely, there are also examples where cysteine oxidation prevents functional aggregation rather than trigger it. For example, yeast ataxin2 spontaneously forms liquid-like droplets that can convert into β-amyloid fibrils. Oxidation of ataxin1 regulates this process by melting the droplets, a process that can be reversed by methionine sulfoxide reductases [80]. Phase-separated ataxin2 is an inhibitor of TORC1 during respiratory growth, thereby stimulating autophagy. Reactivation of TORC1 under oxidizing conditions though regulation of ataxin2, in combination with nutrient starvation thereby allows the coupling of mitochondrial function to TORC1-mediated metabolism [81].

This combination of findings suggests a more general mechanism, where reversible redox-regulated protein aggregation directly dictates protein activity. It also has significant implications for our understanding of aggregated proteins, which are not solely a waste product of misfolded proteins but rather a temporary conformation linked to protein activity.

### Cysteine oxidation-driven protein aggregation in disease

Cysteine oxidation is also involved in toxic protein aggregation. This is illustrated by the amorphous aggregation of γ-crystallins, known to cause cataract. Reduced antioxidant capacity induces the formation of an intramolecular disulfide bond between C32 and C41, which is both necessary and sufficient to induce irreversible aggregation through the destabilization of the N-terminus [82,83]. This is especially interesting in an age-related context since the reducing capacity of the eye diminishes with age [84].

Another detrimental type of aggregation is caused by mutations resulting in an uneven number of cysteines in the transmembrane receptor NOTCH3. NOTCH3 contains a highly conserved even number of cysteines forming disulfide bridges that maintain structural integrity. Any unpaired cysteine stimulates multimerization and aggregation through the loss of a structural disulfides and exposure of an oxidation-prone cysteine that can form non-native disulfide [85,86]. Aggregated NOTCH3 accumulates in the vascular wall, leading to the rare systemic vasculopathology CASADIL.

Oxidation can also occur on other amino acids like methionine which can drastically alter protein structure. In this way, oxidation of a surface-exposed vital residue results in misfolding and aggregation. Several proteins follow this order of events. Examples include: GAPDH [87], γ-synuclein [88], interferon β1a [89], human growth hormone [90], κ-casein [91], FasL [92], transthyretin [93], apolipoprotein AI [94], AMPK [95], PrP [96] and huntingtin [97].

Interestingly, oxidation also triggers the aggregation of direct redox modulators. Cu,Zn-superoxide dismutase (SOD1) is an enzyme important for the dismutation of superoxide to H_2_O_2_ and O_2_, and its misfolding and fibrillization plays a crucial role in the aetiology of the familial form of ALS. The enzymatic product of SOD1, H_2_O_2_, can directly oxidize the surface-exposed C111 in SOD1 which triggers its amyloid fibrillization. In a different manner, higher concentrations of H_2_O_2_ overoxidize C111 to SO_2/3_H causing amorphous aggregation [98–102]. Additionally, mutations causing conformational changes in SOD1 expose its four cysteines, making the structural disulfide bond between C57 and C146 accessible to reduction by the TRX and GSH-GRX systems [101]. Subsequent oxidation leads to the formation of non-native disulfides that cause the formation of insoluble SOD1 multimers and aggregates. Contributing to the cytotoxicity of SOD1 aggregates, SOD1 oxidation has been shown to co-occur with cytoplasmic mislocalization and fibrillization of TAR-DNA-binding protein TDP-43, thereby inducing apoptosis [102].

Besides facilitating aggregation, cysteine oxidation can also prevent it. This is evident for human amylin (hIAPP), which forms a disulfide bridge between C2 and C7 upon oxidation. Oxidized hIAPP stabilizes an α-helical structure at the N-terminus, protecting the peptide from amyloid formation and safeguarding its activity in insulin and glucagon secretion as well as reducing food intake and gastric emptying [103,104]. A similar mechanism has been published for β-microglobulin and endostatin, for which two disulfide bonds guard its native folded conformation [105–107].

Furthermore, oxidation does not only trigger aggregation. Post-aggregation oxidation of proteins seems to be widespread, changing the structural conformation of established aggregates of proteins including huntingtin and β-microglobulin [106,107]. Besides providing an explanation for the abundance of oxidative modifications found in aggregate deposits, this finding suggests that insoluble aggregates are not inert protein disposals but can still alter their structure and interactions due to redox-dependent post-aggregation modifications. This view is supported by the concept that distinct structures of aggregated proteins also cause distinct phenotypes [108,109]. Targeting post-misfolding oxidation might also offer therapeutic opportunities. For example, thiol-reactive compounds can force refolding and reactivation of mutant p53 tumour suppressor (be it direct or indirect) [110,111], a concept that could be meaningful in proteinopathies.

**Figure 2.**
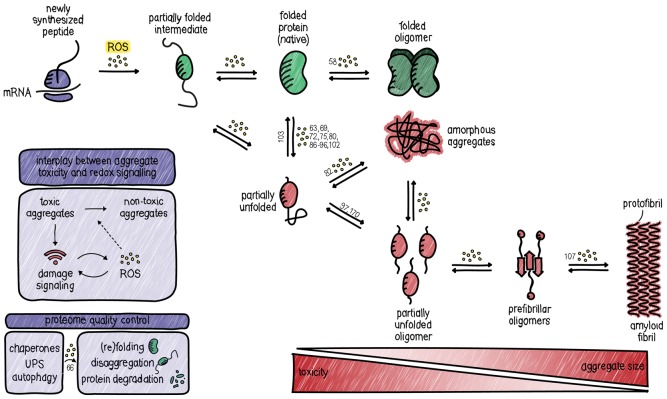
The proteostasis network. Cells employ several mechanisms to maintain proteins integrity and minimize non-native or harmful protein conformations. Mechanisms are in place for proteome quality control. Redox signalling participates in proteostasis by modulating the folding, misfolding, (dis)aggregation and the extent of toxicity of protein aggregates. References to examples of the various steps are indicated in numbers. Note that in some cases it is not clear from the literature what exact step in aggregation is affected, or whether multiple steps are affected, and in this case, the reference is denoted at the first transition from native to partially unfolded.

The previously described cases of protein aggregation and dysfunction induced by cysteine oxidation seem in line with a causal role for ROS in proteinopathies. However, it has been suggested that there is an inverse correlation between the size and toxicity of aggregates, meaning that an increase in size from toxic oligomeric intermediates to insoluble aggregates means a decrease in detrimental effects (Figure 2). In that line of reasoning, stimulating the formation of insoluble aggregates might be a cellular means to confine toxic oligomers. In accordance with this, several types of smaller soluble oligomeric aggregates (including Aβ, α-synuclein and huntingtin) actually impair the 20S/26S proteasome by stabilizing its closed conformation [37]. This could mean that the formation of the more toxic soluble oligomers from natively folded proteins triggers a unidirectional switch, forming insoluble aggregates due to the inability of the proteasome to break down misfolded proteins. Redox signalling-dependent protein oxidation could, therefore, be a facilitator of aggregation and actually partake in the proteotoxic stress response. In support of this, it was shown that promoting the formation of large insoluble aggregates is protective against amyloid-induced ROS production [112].

In summary, the cellular redox environment and protein aggregation show a strong association. With examples of protein oxidation both inducing and preventing aggregation (summarized in Table 1), having functional as wells as pathological consequences and occurring both before and after aggregation, there seems no unifying role for protein oxidation in protein aggregation. However, many proteins do seem to follow the same sequence of events upon oxidation, which includes a partial unfolding and subsequent aggregation step.

**Table 1.** Summary of redox-regulated protein aggregates

Protein	Abbreviation	Normal function	Involved residue (Cys)	Modification	Consequence(s)	Type of aggregation	Related pathologies	Reference
AMP-activated protein kinase	AMPK-alpha	Energy metabolism	C130 and C174	Disulfide	Inactivation	Soluble aggregates	Cardiopathologies, energy starvation	[95]
Apolipoprotein A-I	APOA1	Cholesterol transport	Methionines	Methionine oxidation	Partial unfolding, fibrillization and inactivation	Amyloid	APOA1 amyloidoses and atherosclerosis	[94]
APP/Amyloid-β	Aβ	Unknown	M35	Methionine oxidation	Required for pro-oxidative activity	Amyloid	Alzheimer's	[119]
Ataxin-2	ATXN2	TORC1 inhibition	Methionines	Methionine oxidation	Oxidation reverses aggregation, functional regulation of activity	LLPS, amyloid	Spinocerebellar atrophy, ALS	[80]
Cellular prion protein	PrP^C^ and PrP^SC^	Synaptic function	C179 and C214	Disulfide exchange	Reduction of PrP^C^ induces aggregation of PrP^SC^ polymer	Amyloid	Transmissible spongiform encephalopathies	[96]
Cyclin-dependent kinase inhibitor 2A	p16^INK4A^	Cell cycle regulation, senescence	C72	Homodimerisation	Inactivation	Amyloid	Cancer	[69]
Endostatin	COL18A1	Angiogenesis inhibition	C33, C135, C165 and C173	Disulfide	Prevents aggregation	Amyloid	Alzheimer's	[105]
Fas ligand	FasL	Apoptosis, inflammation	Methionines	Methionine oxidation	Multimerization and aggregation enhanced biological activity	Unknown	Acute lung injury	[92]
Glyceraldehyde-3-phosphate dehydrogenase	GAPDH	Glycolysis	M46	Methionine oxidation, disulfides	Local conformational change promotes disulfide cross-linking and aggregation	Amyloid	Alzheimer's, motor neuron disease	[87]
Growth hormone (recombinant)	hGH	(Therapeutic protein production)	M14 and M125	Methionine oxidation	Lower stability	Unknown	GH deficiency	[90]
human Islet Amyloid Polypeptide	hIAPP	Insulin/glucagon secretion, gastric emptying	C2 and C7	Disulfide	Prevents aggregation	Amyloid	Type 2 diabetes	[103]
Huntingtin	HTT	Unknown	M8	Post-aggregation methionine oxidation	Controls interaction between aggregates	Amyloid	Huntington disease	[106]
			C115 and C119	Disulfide mediated oligomerization	Oxidation-dependent soluble toxic oligomers, slower clearance	Soluble oligomeric aggregates		[97]
Interferon-β1a (recombinant)	IFNβ1a	(Therapeutic protein production)	Many residues (M, H, F, W, Y)	Cross-linking	Lower stability	Unknown		[89]
Mitochondrial GrpE protein homologue	MGE1	Proteostasis, HSP70 cochaperone	M155	methionine oxidation	Inactive HSP70 but targeting it to unfolded proteins	Amyloid	Myopathies	[141]
Sequestosome-1	SQSTM1/p62	Autophagy	C105 and C113	Disulfide mediated oligomerization	More autophagy, cell survival	Insoluble aggregates, LLPS	ALS	[170]
Superoxide dismutase 1	SOD1	Dismutation of superoxide	C111	Disulfide-linked dimerization	Oligomerization and subsequent fibril formation	Oligomeric, amyloid and amorphous	ALS	[102]
Transthyretin	TTR	Thyroid hormone binding	C10, M1 and M13	Cysteine sulfonic acid, methionine sulfoxide	Tetramer dissociation and aggregation	Amyloid	Senile systemic amyloidosis	[93]
Tryptophan hydroxylase 2	TPH2	Serotonin biosynthesis	Any out of 13 cysteines	Disulfide, cross-linking	Misfolding, intra- and intermolecular disulfide bond formation, protein inactivation	Unknown, disulfide cross-linked oligomers	Parkinson's	[72]
Vinisin-like protein 1	VSNL1	Calcium sensing	C187	Disulfide-linked homodimerization	Reduced levels of functional protein	Amyloid, disulfide cross-linked oligomers	ALS, AD	[75]
y-synuclein	SNCG	Neurofilament network integrity	M38 and Y39	Oxidation-dependent oligomerization	Aggregation and seeding for α-synuclein aggregation	Amyloid	Parkinson's	[88]
β2-microglobulin	β2M	MHCI light chain	C25 and C80	Disulfide reduction, disulfide exchange, post-aggregation oxidation	Aggregation, post-aggregation stabilization	Amyloid	Hemodialysis-related amyloidosis	[107]
γ-crystallins	CRYG	Lens transparency	C32 and C41	Intramolecular disulfide	Destabilizes its N-terminal domain, stabilizes an intermediate which is prone to aggregation	Amorphous	Cataract	[82]
κ-casein	CSN3	Milk protein	M95 and M106	Methionine oxidation	Increased aggregation, increased toxicity	Amyloid	Corpora amylacea	[91]

## Effects of aggregation on the cellular redox state

Protein aggregation can also modulate the cellular redox state, and, as mentioned, this could either be part of the proteotoxic stress response, cellular dysfunction as a result of the accumulation of protein aggregates, or both. For example, based on thiol-disulfide redox couples and redox sensors it has been determined that under basal conditions mitochondria, the cytosol and nucleus are in a relatively reduced state as compared with the ER. This reverses radically when protein aggregation induces proteasomal dysfunction, resulting in a more oxidizing cytosol and reducing ER [60,113]. S-nitrosylation could be a possible contributor to this change due to the overactivation of the *N*-methyl-d-aspartate receptor (NMDAR, an inducer of neuronal nitric oxide synthase (nNOS)) or Aβ-dependent iNOS activation in neurons and glial cells of AD patients [114–116]. A shift to a more oxidizing cellular redox state upon proteotoxic stress is further supported by many reversible cysteine modifications that change in models for proteinopathies like AD [117,118].

A more direct explanation for the redox changes that occur upon aggregation can be found in the direct redox-dependent aggregation of redox modulators. As discussed above, SOD1 aggregation is directly triggered by oxidation of C111, thereby inactivating its function. This leads to the accumulation of superoxide at the expense of H_2_O_2_, where the former is more associated with random damage and the latter with redox signalling.

Another effect of aggregation on the cellular redox state is exemplified by the transmembrane Aβ protein precursor (AβPP), which is the precursor for the archetypical amyloidogenic Aβ peptide and the main component of amyloid plaques in brains of AD patients. Interestingly, monomeric Aβ is suggested to have an antioxidant activity by hydrophilic chelation of transition metals, thereby preventing lipoprotein oxidation [119–122]. However, this chelation of metals also promotes the aggregation of Aβ, and redox-active metal ions like Cu(II) and Fe(III) can catalyze the production of ROS when bound to aggregated Aβ. Aβ binding results in the reduction of the metal's oxidation state, which then converts O_2_ into H_2_O_2_, superoxide and hydroxyl radicals via Fenton chemistry [123–126]. Hence, the binding of Aβ to metals changes its properties from an antioxidant to a pro-oxidant [120]. ROS-induced o,o′-dityrosine covalent cross-linking then catalyzes further aggregation of Aβ [127]. Interestingly, a similar interplay between metals and oligomers has been reported for α-synuclein [128], pointing potentially at a more general mechanism in which misfolded oligomeric proteins in conjunction with metal ions induce the production of ROS. Metal concentrations, aggregation as well as ROS production all increase with age and even without knowing the exact cause, the consequence is faster neurodegeneration [129].

Furthermore, many aggregates can directly cause mitochondrial dysfunction, resulting in metabolic stress, enhanced ROS production and eventually cell death. For example, amyloid oligomers are well-known for their permeabilization of membranes, which is considered a main toxicity event. A rapid influx of intracellular Ca^2+^, as well as the direct permeabilization of the mitochondrial membrane, causes an increase in mitochondrial ROS production [130–133].

When unfolded proteins accumulate in the ER, a condition called ER stress triggers the unfolded protein response (UPR) in an attempt to restore homeostasis. Protein aggregates like oligomeric Aβ have been shown to trigger ER stress and the UPR [134,135]. Prolonged ER stress is known to evoke intracellular ROS production at the ER through several mechanisms. These include overactivation of Ero1 oxidoreductases through a futile cycle of forming and repairing mismatched disulfides, thereby producing H_2_O_2_ and oxidizing GSH, respectively [132,136,137]. In addition, ER stress causes superoxide production though activation of NOXs and the release of Ca^2+^ which increases electron leakage from mitochondria (for a review see [138]). Hence, since ROS are both a trigger and a consequence of ER stress, this further aggravates the imbalance accompanying ER stress [139].

## Redox regulation of aggregate clearance

To cope with the challenges caused by protein aggregation, cells are equipped with several mechanisms aimed at the clearance of misfolded proteins and aggregates, which themselves are also shown to be redox-regulated, adding another level of the complex interdependency of proteostasis and ROS.

Besides their role in folding of newly synthesized proteins, molecular chaperones are also part of this cellular disaggregation machinery. There are many types of chaperones, but a general initial step seems to be the initial coverage of aggregates with HSP70 [140]. An example of the redox regulation of HSPs is through the reversible oxidation of MGE1, a mitochondrial nucleotide exchange factor of HSP70, on M155. The consequential structural change from an active homodimer to monomer leads to MGE1 amyloid formation, which prevents the activation of HSP70. Interestingly, oxidized MGE1 is suggested to increases the binding affinity of the inactive HSP70 for unfolded substrates. As HSP70 is essential in protein folding and proteostasis, MGE1 might act as an initial sensor of protein aggregation, priming the chaperone system for resolution of protein aggregates [141,142].

Peroxiredoxins are more unconventional chaperones. With their abundance and exceptional reactivity to H_2_O_2_, peroxiredoxins are important H_2_O_2_ scavengers. Their highly conserved active site consists of two catalytic cysteine residues. Besides their antioxidant activity, peroxiredoxins are known for their ability to oxidize protein thiols by a redox relay as well as for their chaperone activity [143]. Assembled as a high molecular mass complex, peroxiredoxins can form ring-like chaperone structures with holdase activity that bind and prevent aggregation of unfolded proteins [144,145]. Among others, hyperoxidation of the peroxiredoxin catalytic cysteine particularly stimulates an oligomeric chaperone structure, whereas glutathionylation inhibits it [146–149]. Thereby peroxiredoxins can sense high and low ROS levels and switch their function accordingly from antioxidant and redox signalling mediator to chaperone.

Redox control of the UPS is complex, with studies claiming both inhibitory and activating effects. In general, the 20S, but not 26S proteasome is thought to specifically degrade oxidized proteins [150–152]. Elevated ROS and mitochondrial dysfunction shift the proteasome population from 26S to 20S, thereby adapting to the proteolytic need for clearance of oxidized substrates [153]. In support of this, proteasomal degradation has been shown to be stimulated >10-fold upon exposure to H_2_O_2_ or superoxide, while simultaneously abolishing 26S-mediated degradation [154–156]. The proteasome is also subject to direct redox modifications. For example, the 20S proteasome can be glutathionylated, resulting in its opening and activation [156,157]. Thus, under oxidizing conditions, the 20S proteasome is stimulated to clear oxidized substrates, but this shift away from 26S permits the accumulation of otherwise misfolded proteins. This, however, has been debated by the notion that a high NAD^+^/NADH ratio, correlating with an oxidizing cellular state, opens and activates the 26S proteasome [158,159]. Also, the lipid peroxidation byproduct HNE was found to inhibit proteasomal activity for the breakdown of oxidized proteins [160,161]. In summary, oxidizing conditions result in a shift to the 20S proteasome, accompanied by a possible decrease in 26S activity. This might favour the accumulation of non-oxidized misfolded proteins. Moreover, the interaction between the cellular redox state and the proteasome is bidirectional. The blockage of the proteasome, for instance, leads to an increase in ROS, which in turn might cause a vicious cycle of protein oxidation and aggregation [162,163].

It has been suggested that both aggregation and cellular redox state are coupled through their regulation of autophagy [164]. Autophagy is thought to be activated in more oxidizing conditions [165–168], although this might not always be straightforward as glutathione reductase loss, resulting in oxidizing conditions, was recently shown to suppress autophagy and enhance aggregation [169]. The recruitment of ubiquitinylated substrates to autophagosomes is mediated by receptors like SQSTM1/p62. Interestingly, components of the autophagy system have also been found to aggregate in a redox-dependent manner themselves. For example, oxidation of p62 at C105/113 in the N-terminal disordered region causes oligomerization and subsequent aggregation of p62. This stimulates autophagy in response to ROS, possibly in an attempt to maintain cellular homeostasis [170]. In addition, aggregated p62 occupies the NRF2-binding site in KEAP1, allowing the stabilization of NRF2. NRF2 nuclear translocation causes the expression of antioxidant- and stress response genes, among which p62 itself [171]. Redox-dependent p62 aggregation, therefore, ensures a robust stress response involving both autophagy and antioxidant response. Accordingly, mutations perturbing the redox-sensitivity of the NRF2 pathway are linked to ALS. Of note, mutant KRAS induced lung tumours in mice have been shown to depend on the NRF2 pathway for their survival and outgrowth [172], likely because it facilitates antioxidant- and autophagy-dependent clearance of cancer-associated proteotoxic and metabolic stress.

Taken together, protein aggregation has been shown to modulate ROS levels in several ways. Some of the increases in ROS production upon the gradual accumulation of aggregates might, therefore, be an attempt to regain homeostasis through modulation of the stress response. This eventually reaches a turning point when ROS reaches toxic levels and triggers a stress response on its own [2]. The notion that oxidation by ROS can facilitate protein aggregation and that amyloids themselves can trigger ROS production could in principle also constitute a feed-forward loop in which a small change in either ROS or protein aggregation could rapidly lead to a toxic cellular catastrophe, making redox-regulated protein aggregation an irreversible process [173]. But extensive random damage induced by ROS production either from an overactive stress response or from gross cellular dysfunction resulting in for instance lipid peroxidation might also have an evolutionary benefit and serve to actively eliminate damaged cells through the induction of ferroptosis [174].

## Conclusions

In this review, we have discussed the reciprocal regulation of redox signalling and protein aggregation. In short, oxidation of specific residues causes conformational changes and causes partial unfolding of a protein. This exposes residues that can participate in non-native interactions. Further structural rearrangements drive the oligomerization and subsequent formation of insoluble aggregates. We can distinguish two types of cysteine residues here: structural residues that form disulfide bonds for correct protein folding, and cysteines that can be reversibly oxidized which serve as important signalling switches. When reversible disulfide bond formation changes the protein structure such that it directly corresponds to protein function, it can be both a structural and a regulatory residue. Although the underlying processes seem somewhat similar, the functional consequences of redox-dependent protein aggregation are not. Besides simply resulting in toxic protein aggregates, oxidative aggregation can result in reversible (in)activation of a protein which allows regulation. This can alter protein function and may serve as a protective or pro-survival mechanism. Although little is known about functional aggregation, it is thought that most aggregates are actually reversible, benefiting the regulatory possibilities. This is also reflected in the suggestion that insoluble aggregates are not inert protein disposals but can still alter its structure and morphology according to redox-dependent post-aggregation modifications and that distinct structures of aggregated proteins also cause distinct phenotypes.

On the other hand, there is the hypothesis that ROS-induced aggregation might be a cellular strategy to clear the more toxic soluble aggregates. Many of the examples we discussed in this review support this; they are part of a positive feedback system in which high ROS levels promote aggregation and the aggregates themselves promote ROS production. In this way, redox signalling creates a bistable switch between functional proteins and insoluble aggregates without the accumulation of toxic intermediates. Therefore, modulating ROS levels to promote rather than inhibit aggregation could be a more sensible therapeutic approach.

**Figure 3.**
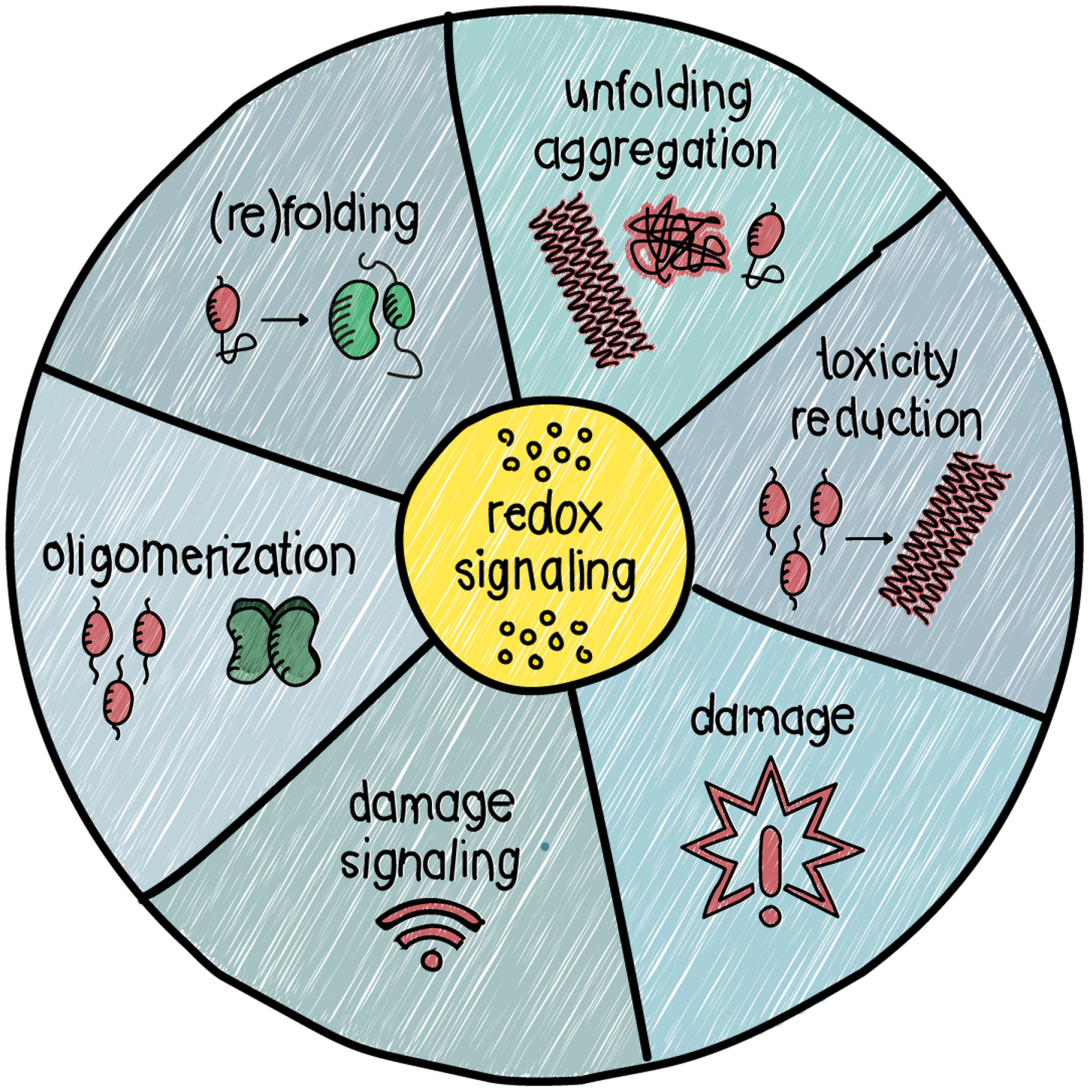
Redox regulation of proteostasis. Redox signalling modulates proteostasis in many ways. Among its roles are the regulation of protein folding, unfolding/aggregation, toxicity reduction and damage response.

Almost all the processes involved in protein aggregation are redox regulated (Figure 3). However, it is hard to determine whether altered redox signalling is a cause or consequence of protein aggregation. Often times it seems like it is both: altered redox signalling favours aggregation, but the mutual amplification of the systems also provides a feed-forward loop, consequently altering the cellular redox state. One clue might lie in the hypothesis stating that H_2_O_2_ is produced as a signalling molecule in response to damage, such as an accumulation of aggregates. A gradual increase in aggregation is accompanied by an increased H_2_O_2_ production, which intensifies over time. Eventually, the H_2_O_2_ might reach toxic levels, which itself may lead to damage. Understanding whether H_2_O_2_ is produced as a stress response in order to regain homeostasis might change our view of proteinopathies.

Why aggregation is so much more prominent in aged individuals is not entirely clear. The age-dependent increase of aggregation, oxidation and mitochondrial dysfunction, together with a decline in multiple aggregation clearance systems could cumulatively cause a destabilizing environment from which protein aggregates can no longer recover [175,176]. The diverse effects of oxidative aggregation on proteins suggest that in order to regulate proteins by oxidative aggregation, the reversibility of the process is essential in some cases. Little is known about the reversibility of oxidized aggregates. For example, when the reducing capacity of a cell is restored, can the aggregates fall apart into functional monomers again by reduction of the oxidative modifications? It is also not clear if and how cellular systems to clear protein aggregates such as the proteasome and autophagy can distinguish between ‘functional’ aggregates that are a temporary, regulated protein state, and toxic protein aggregates that need clearance.

## Perspectives

- *Importance of the field*: Both redox signalling and correct protein folding are essential for maintaining healthy cellular homeostasis. Accordingly, loss of proteostasis and ROS production as a consequence of mitochondrial dysfunction have been described as hallmarks of ageing. This becomes especially clear in proteinopathies, where both aberrant redox signalling and protein aggregation are associated with severe neurodegenerative problems.
- *Summary of the current thinking*: Collectively, these studies outline the complex relationship between ROS, redox signalling and proteostasis, where cause and consequence are often hard to differentiate. Whereas protein oxidation can trigger aggregation, increases in H_2_O_2_ production upon the gradual accumulation of aggregates might be a stress response by itself. A small change in redox state or aggregation can in this way rapidly lead to a feed-forward loop, making redox-regulated protein aggregation an irreversible process.
- *Future directions*: Going forward, it is important to better understand whether or not H_2_O_2_ is produced as a signal in response to proteotoxic stress in order to restore homeostasis, and whether increased H_2_O_2_ levels promote aggregation as a cellular strategy to clear the more toxic oligomeric aggregates. Understanding the multifaceted mechanisms regulating protein aggregation will pave the way for novel therapeutic windows to combat proteinopathies.

## Abbreviations

ALS: amyotrophic lateral sclerosis
APRs: aggregation prone regions
AβPP: amyloid-β protein precursor
C: cysteine
DUOX: dual oxidase
H_2_O_2_: hydrogen peroxide
IDRs: intrinsically disordered regions
iNOS: inducible nitric oxide synthase
LLPS: liquid–liquid phase separation
M: methionine
NMDAR: *N*-methyl-d-aspartate receptor
nNOS: neuronal nitric oxide synthase
NOX: NADPH oxidase
O_2_^•−^: superoxide anion
OH^•^: hydroxyl radical
ROS: reactive oxygen species
SOD1: Cu,Zn-superoxide dismutase
TPH2: tryptophan hydroxylase 2
UPR: unfolded protein response
UPS: ubiquitin-proteasome system
VSNL1: visinin-like protein-1
